# Infrared Stimulated Luminescence of Ce^3+^ Doped YAG Crystals

**DOI:** 10.3390/ma15238288

**Published:** 2022-11-22

**Authors:** Paweł Bilski, Anna Mrozik, Wojciech Gieszczyk, Sergiy Nizhankovskiy, Yuriy Zorenko

**Affiliations:** 1 Institute of Nuclear Physics Polish Academy of Sciences, Radzikowskiego 152, 31342 Kraków, Poland; 2 Institute for Single Crystals, National Academy of Sciences of Ukraine, av. Nauki 60, 61178 Kharkiv, Ukraine; 3 Institute of Physics, Kazimierz Wielki University in Bydgoszcz, Powstańców Wielkopolskich Str. 2, 85090 Bydgoszcz, Poland; 4 Oncology Center, Medical Physics Department, Romanowskiej 2, 85796 Bydgoszcz, Poland

**Keywords:** optically stimulated luminescence, dosimetry, garnets, IRSL

## Abstract

In this study, the infrared optically stimulated luminescence (IRSL) of single crystals of Ce^3+^ doped yttrium aluminum garnet (YAG) was investigated for the first time. It was found that infrared stimulation of these crystals, following previous exposure to beta radiation, produces a strong luminescence signal. The highest luminescence efficiency was exhibited by the YAG crystal with 0.1% of Ce. With this crystal, it was possible to measure as low doses as 0.1 mGy. Moreover, IRSL is mainly related to the TL peak at a relatively high temperature of c.a. 175 °C, which leads to quite good stability of the signal in time. These properties create good prospects for potential applications of YAG:Ce in dosimetric radiation measurements

## 1. Introduction

Garnets doped with rare-earth elements, and among them especially yttrium-aluminum garnet Y_3_Al_5_O_12_ (YAG), are well-known optical materials of various applications. YAG crystals doped with Nd^3+^ or Er^3+^ have been used for years as host materials in solid-state lasers [1,2]. YAG:Ce^3+^ was applied for electron-beam tube applications [3]. It is used in white light-emitting diodes (WLEDs) as a photoconverter [4]. The crystals and single crystalline films of this garnet are also widely used as scintillators for ionizing radiation measurements and imaging [5]. Finally, the YAG:Ce crystals possess suitable thermoluminescent (TL) properties for dosimetric application [6,7].

Recently, it was found that scintillating materials of the garnet family may also exhibit interesting properties of optically stimulated luminescence (OSL), which potentially may be exploited for ionizing radiation dosimetry. Firstly, a red-stimulated luminescence was found in lutetium-aluminum garnet [8], and then, in our previous paper, we described infrared-stimulated luminescence of gadolinium aluminum-gallium mixed garnet Gd_3_Ga_x_Al_5−x_O_12_ (GGAG:Ce) [9]. When stimulated with IR at about 870 nm, the previously irradiated GGAG:Ce crystals exhibit very strong luminescent emission in the range of 500–600 nm, which is typical for Ce^3+^ emission in garnets. OSL is the method more and more widely used in dosimetry [10,11], but IR stimulation (IRSL) was not yet exploited for this purpose. Such stimulation is so far used almost exclusively for the dating of geological sediments and archeological artifacts (mainly measurements of luminescence of feldspar minerals). OSL method is especially useful for dosimetry if the wavelength of the stimulation light is longer than the emission wavelength. Such configuration enables avoidance of usually high fluorescent backgrounds, which is always present if stimulation is completed at a shorter wavelength than emission. For that reason, typical OSL dosimetry materials (Al_2_O_3_:C, BeO) are stimulated with blue or green light and the measurement window is in the UV range. The application of IR for stimulation allows for making use of the Ce^3+^ emission in garnets, opening this wide family of well-known luminescence crystals for potential use in dosimetry. In the present work, we have continued this approach, investigating the IR-stimulated luminescence of Ce^3+^ doped YAG crystals in perspective of their use as passive radiation dosimeters.

It is worth noting also here that, the YAG:Ce crystals, grown from a melt at high (1930 °C) temperature, possess various types of host defects [12,13,14,15,16,17]. The most known of them are the antisite Y_Al_ [18,19,20,21] and charged oxygen vacancies (F^+^ and F centers) [22,23,24] as well as the various aggregates of mentioned centers [22,25]. In general, some of these clustering centers may have excitation (absorption) spectra in the IR range. Taking into account that the intensity of IR OSL usually depends on the dopant concentration [8,9], the formation of pair centers with the participation of YAG host defects and Ce^3+^ ions was not excluded as well. Identification of the nature of such centers is a hot topic and such research with the use of theoretical calculation [15,16], advanced optical methods [19,20], and radio-spectroscopic investigations [25] is performed on various garnet compounds.

## 2. Materials and Methods

**Figure 1:**
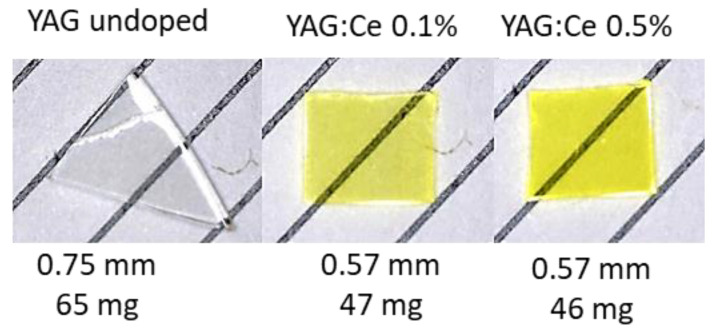
The pictures of the studied YAG crystals, with the data on thickness and mass of the samples. The distance between lines in the background is 3 mm.

The studied YAG crystals were grown by the Horizontal Direct Crystallization (HDC) method at the Institute for Single Crystal in Kharkiv, Ukraine. Crystals with two concentrations of Ce dopant were investigated: 0.1% and 0.5% and compared with a nominally undoped YAG sample (see Figure 1). All crystals were grown in the same conditions. In some measurements, we used for comparison purposes the Ce-doped gadolinium aluminum-gallium garnet Gd_3_Ga_2.5_Al_2.5_O_12_ (GGAG:Ce), which properties were described in the previous publication [9].

The TL and IRSL measurements were carried out with the automated Risø DA-20 TL/OSL reader (DTU Roskilde, Denmark) equipped with an EMI 9235QB photomultiplier. The reader enables both TL and OSL measurements, the latter with blue (470 nm) and infrared (860 nm) stimulation wavelengths (in this work only IR was used). The IR stimulation in the DA-20 reader is realized by 21 LEDs arranged in three clusters, producing a maximum power of approximately 145 mW/cm^2^ at the sample position. In this work, we used always 40% of the maximum power, to avoid an increase in the background caused by the short wavelength tail of the LED spectrum, which was leaking through the optical filter (a BG-39 filter was used in all IRSL and TL measurements). It should be noted that the photomultiplier of this reader has an efficiency decreasing above c.a. 500 nm and nearly null above 600 nm. This affected the obtained results (see Section 3.4). The TL measurements were performed at the rate of 2 °C/s. The DA-20 reader is equipped with a ^9^°Sr/^9^°Y beta source of activity 1.4 MBq providing a dose rate of about 0.05 Gy/s. The IRSL and TL measurements were performed after irradiations with doses in the range of 0.5–2 Gy, with the exception of the spectrally resolved measurements, which required a dose of 100 Gy. For measurements that required low doses (≤10 mGy), an external ^137^Cs gamma-ray source was used. All irradiations were performed at room temperature. The infrared-stimulated luminescence and thermoluminescence emission spectra were measured using the QE pro 00689 spectrometer (Ocean Optics, Orlando, FL, USA) mounted in the DA-20 reader in place of the PMT. The spectrometer allows registering the emission spectra over the wavelength range 200 to 1000 nm with 4 nm resolution. 

## 3. Results

### 3.1. IRSL Decay Curves

**Figure 2:**
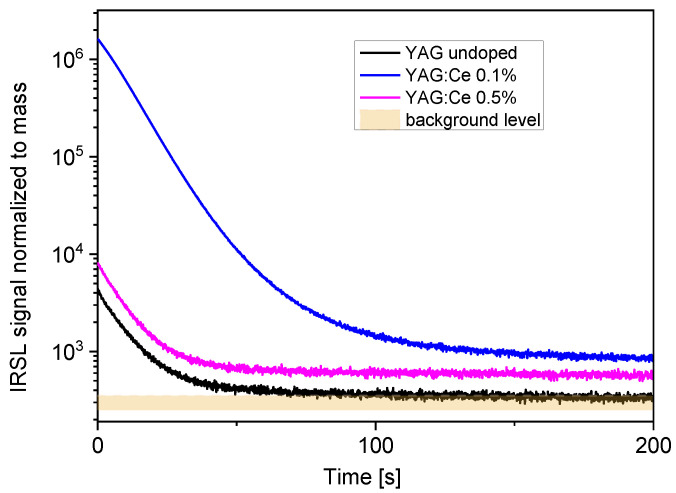
IRSL decay-curves of the studies YAG:Ce crystals. The wider band, corresponding to the background level, illustrates the variation range of the background. Dose 2 Gy.

**Figure 3:**
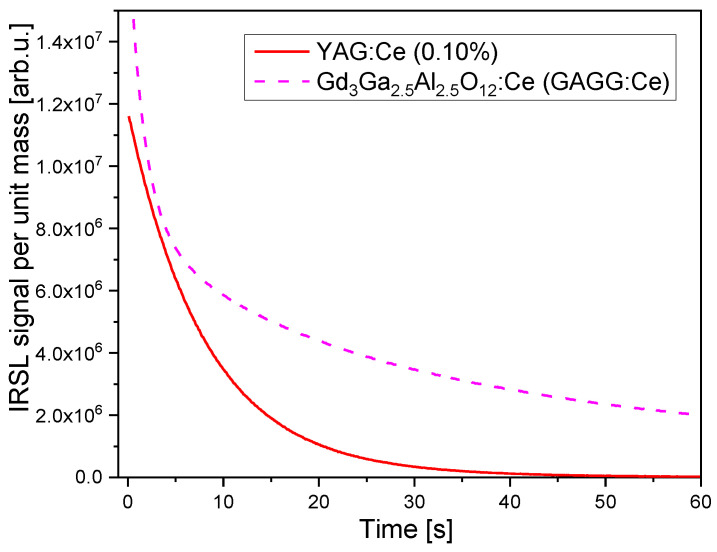
Comparison of IRSL decay-curves of YAG:Ce 0.1% and GAGG:Ce. Dose 2 Gy.

Figure 2 compares the IRSL decay curves of all samples, measured under constant illumination power. The differences in OSL intensity between the samples are very big. The highest intensity was exhibited by the crystal with 0.1% of Ce. The IRSL intensity of the crystal with 0.5% of Ce was nearly three orders of magnitude lower and it is only about twice higher than that of the nominally undoped YAG crystal. The intensity exhibited by the crystal with 0.1% of Ce was quite high—the signal after the 2 Gy dose exceeded the background level by nearly four orders of magnitude, which indicates a potential for applications in radiation dosimetry. It is difficult to compare directly the sensitivity of the studied samples with some standard OSL detectors, as such standards for IR stimulation do not exist. Figure 3 presents the comparison of the YAG:Ce 0.1% with GGAG:Ce crystal described in the previous publication [9]. The IRSL intensity exhibited by YAG:Ce is somewhat lower but on the same level of magnitude. The IRSL decay of YAG:Ce is faster than that of GGAG:Ce, which is advantageous for practical applications. A faster decay means a possibility of a shorter measurement time and a shorter bleaching time before another use of the same detector.

**Figure 4:**
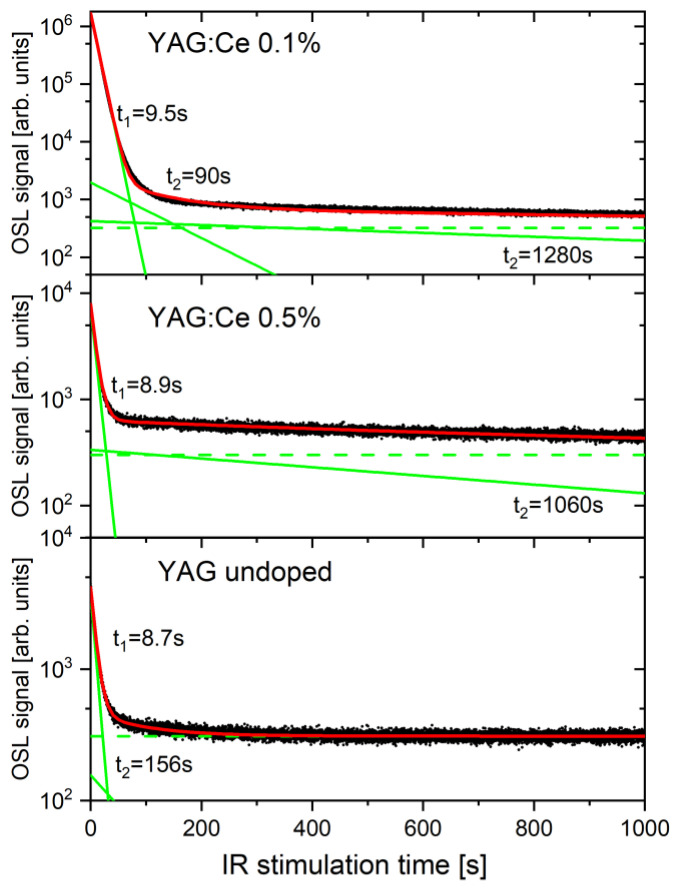
Decomposition of the decay curves of the YAG:Ce crystals into separate components (dose 2 Gy). The measured data (black dots) were fitted with exponential functions (solid green lines) plus a constant background (dash green lines). The sums of the fitted functions are represented by red lines.

The OSL signal measured under constant stimulation can be described by an exponential function of time (assuming first-order kinetics). However, usually, the shape of a decay curve cannot be correctly described by just a single exponent, but rather a sum of exponential functions must be used. Figure 4 presents the results of the analysis of the decay curves registered during 1000 s of IR stimulation. To obtain a satisfactory fit, three exponential components were needed for the crystal with 0.1% of Ce and two components for the remaining samples. In all samples, the dominant is a fast-decaying component with a time constant of around 9 s. The IRSL curves of Ce-doped samples also contain a very slowly decaying component (time-constant > 1000 s). For the crystal with 0.1% Ce, the presence of a medium component (time constant 90 s) is also evident.

### 3.2. Thermoluminescence Glow-Curves

**Figure 5:**
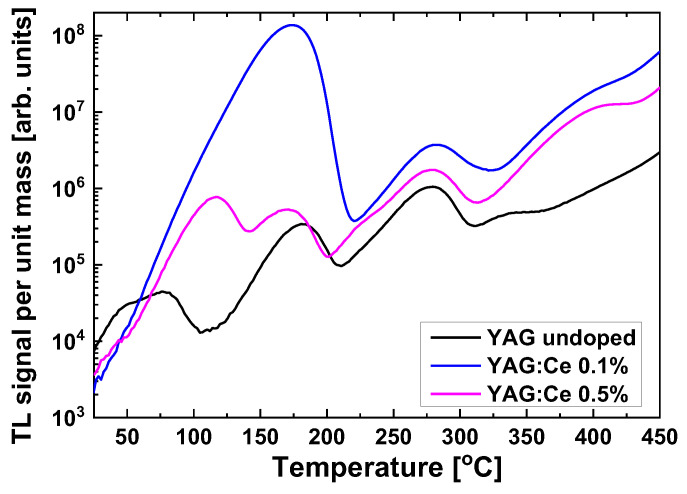
TL glow-curves of the studied YAG:Ce crystals. Dose 2 Gy.

Figure 5 presents the thermoluminescence (TL) glow-curves of the studied samples. TL is a phenomenon related to OSL—trapping sites responsible for OSL are emptied also by thermal stimulation, which usually leads to TL emission. Similarly, as in the case of IRSL, the highest intensity is shown by the sample with 0.1% of Ce. All samples exhibit a peak around 170–180 °C, which is dominant for the glow-curve of YAG:Ce 0.1%. For the two remaining samples, the dominant is the peak located at about 280 °C, which is also present, but relatively much smaller, for the crystal with 0.1% Ce.

**Figure 6:**
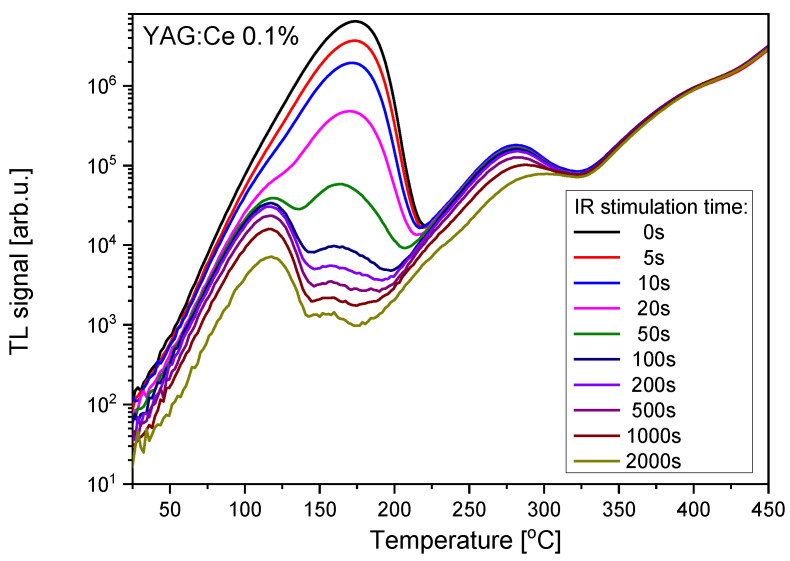
Influence of the variable time of IR illumination on TL glow-curves of YAG:Ce 0.1% crystal. Dose 2 Gy.

**Figure 7:**
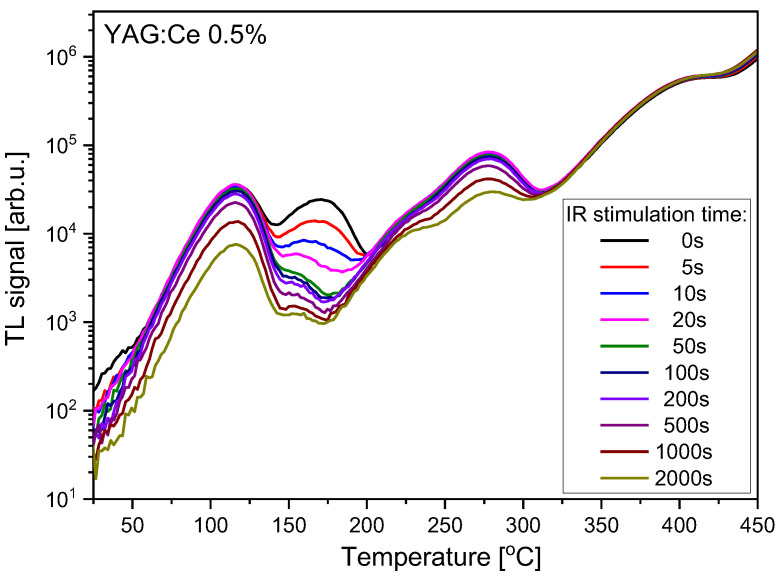
Influence of the variable time of IR illumination on TL glow-curves of YAG:Ce 0.5% crystal. Dose 2 Gy.

**Figure 8:**
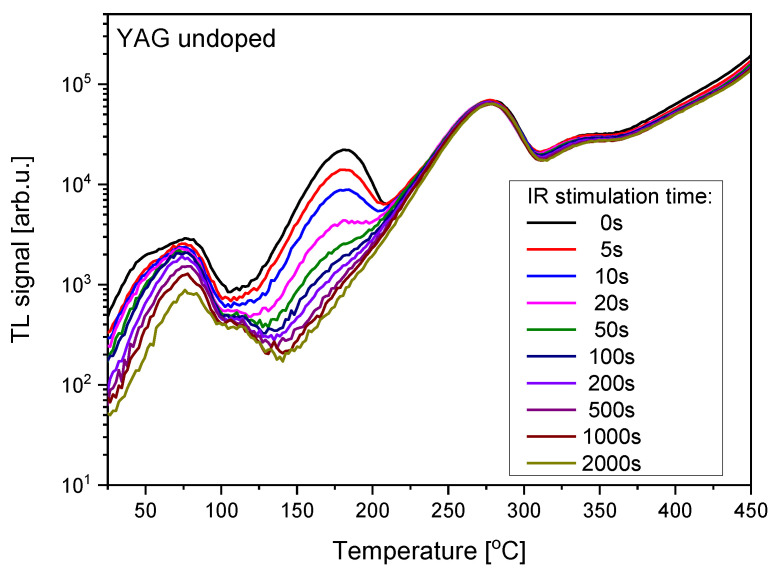
Influence of the variable time of IR illumination on TL glow-curves of undoped YAG crystal. Dose 2 Gy.

Some information about which trapping sites are active during OSL may be obtained by combining TL with OSL, i.e., performing TL measurements on the samples which were previously subjected to stimulation with light for different periods of time. The results of such measurements are presented in Figure 6, Figure 7 and Figure 8. In all cases the IR stimulation mostly reduces the peak located between 150 °C and 200 °C, therefore this peak seems to provide the main contribution to the IRSL signal. The minor contributions come from the low-temperature peaks and, in the case of Ce-doped crystals, from the peak located between 250 °C and 300 °C. The high-temperature peak near 400 °C is not sensitive to IR illumination, similar to the peak at about 270 °C for the undoped YAG.

**Figure 9:**
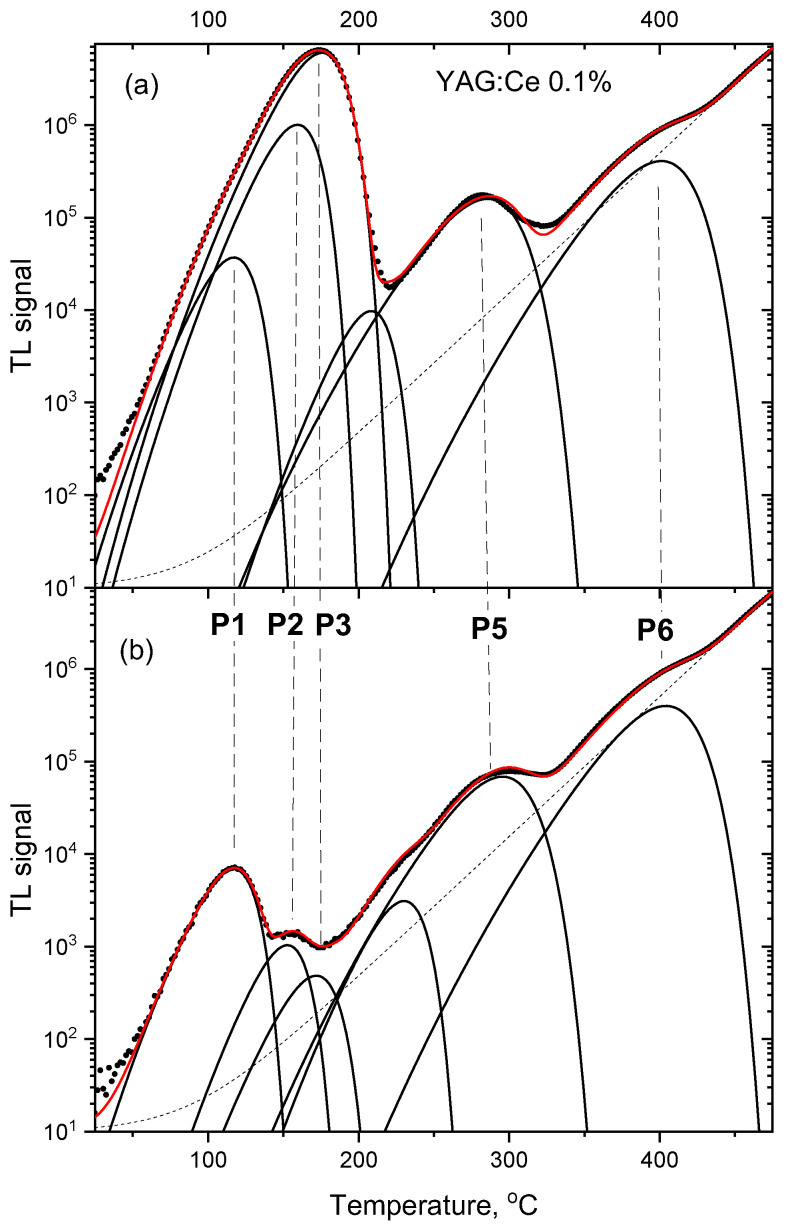
TL glow-curves of YAG:Ce 0.1% crystal, deconvoluted into single peaks. (**a**) no IR illumination, (**b**) after 2000 s of IR illumination. Dose 2 Gy.

**Figure 10:**
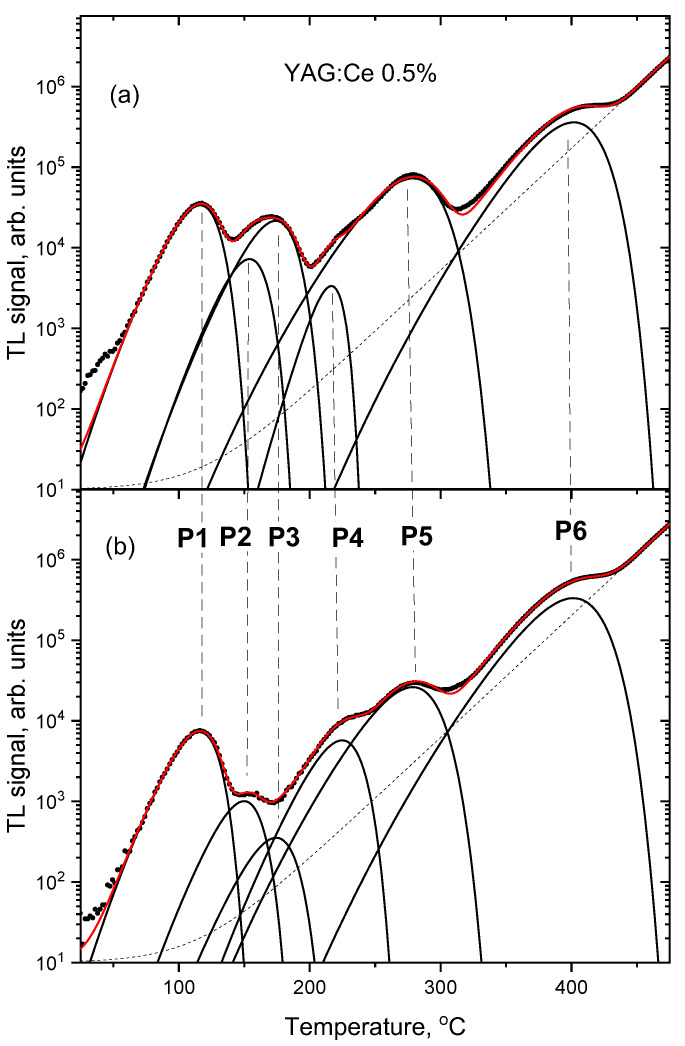
TL glow-curves of YAG:Ce 0.5% crystal, deconvoluted into single peaks. (**a**) no IR illumination, (**b**) after 2000 s of IR illumination. Dose 2 Gy.

**Figure 11:**
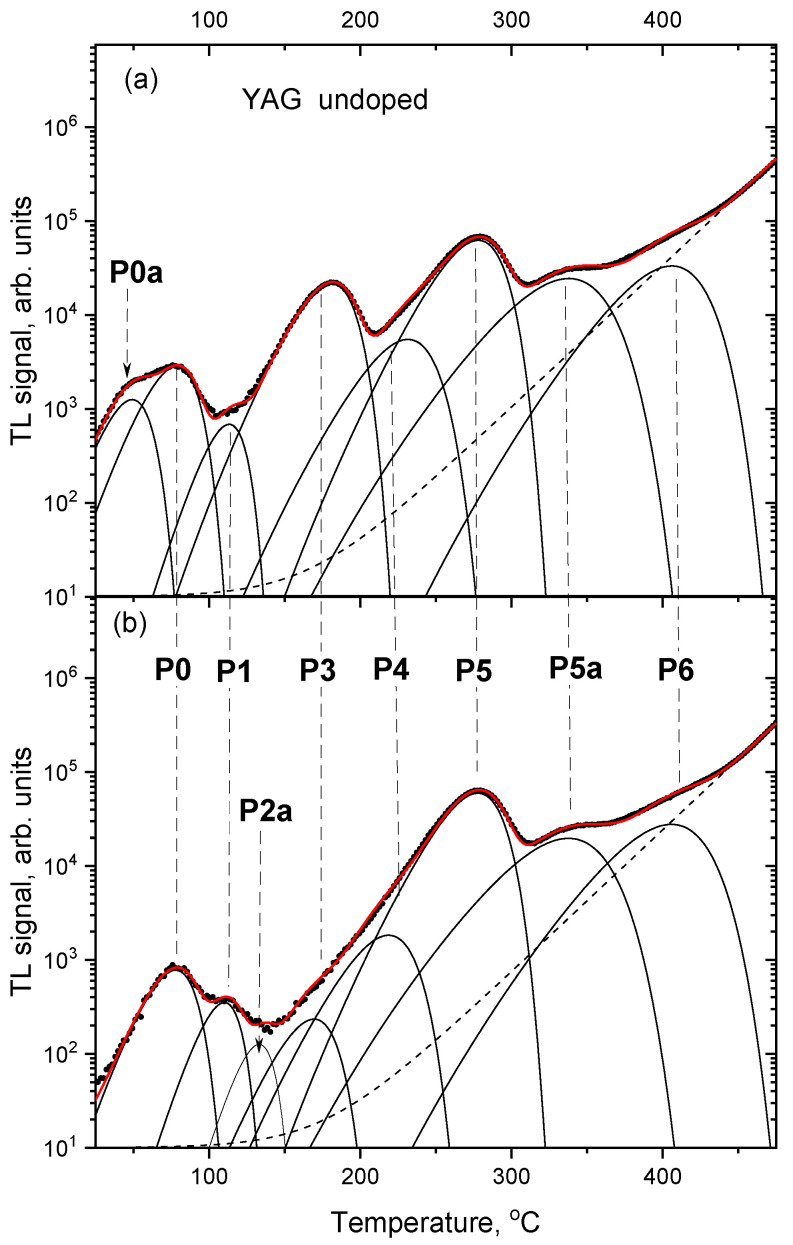
TL glow-curves of YAG undoped crystal deconvoluted into single peaks. (**a**) no IR illumination, (**b**) after 2000 s of IR illumination. Dose 2 Gy.

More in-depth analysis of the TL glow-curves was performed by their deconvolution into separate peaks (assuming peak shapes according to the first-order kinetic model). Figure 9, Figure 10 and Figure 11 compare deconvoluted glow-curves of YAG:Ce crystals after 2000 s IR stimulation, with those not illuminated by IR. It is worth noting, that reduction of the dominant peaks by IR leads to revealing of other peaks, previously unnoticeable. Four peaks seem to be present invariably in all YAG:Ce samples: P1 (located in the range 110–119 °C, depending on a sample and a particular measurement), P3 (167–180 °C), P5 (275–285 °C) and P6 (400–405 °C). As they are present also in the undoped YAG crystal, it seems that they are related to intrinsic defects or/and some unwanted impurities. 

**Figure 12:**
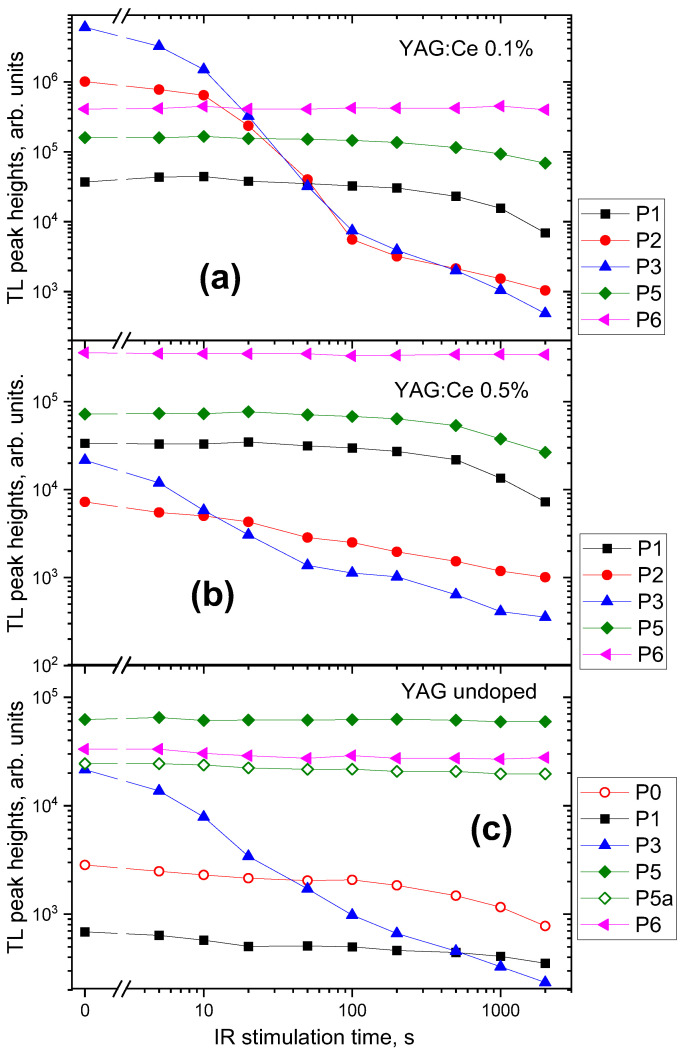
Dependence of the amplitude of the main TL peaks on the IR stimulation time in YAG:Ce 0.1% (**a**), YAG:Ce 0.5% (**b**), and undoped (**c**) crystals, according to the deconvolution with the first-order kinetics. It should be noted, that as peaks P2 and P3 are strongly overlapped, the separation between them is probably biased with large uncertainties.

Figure 12 summarized changes in the fitted amplitude of the main peaks following different times of IR stimulation. It is apparent, that most of the IRSL signal is related to the peaks P2 and P3, which show a steep decrease with the stimulation time. P2 is absent in the undoped YAG, which suggests that it is related to Ce-doping (the peak marked as P2a in Figure 11 seems to be a different entity, as it occurs at a somewhat different temperature and only after the 2000 s of IR). Peaks P2 and P3 are probably responsible for the fast component of the IRSL decay curves. Peak P5 decreases only after long IR illumination, so it is probably responsible for the slow component of the IRSL decay, observed for the YAG:Ce crystals. The peak marked as P5 for the undoped YAG, despite its occurrence at the same temperature range as for other samples, may be related to a different trapping site than corresponding peaks for Ce-doped crystals, as it does not exhibit any decay under IR stimulation. Another peak, marked as P5a is visible in the glow-curve of the undoped YAG, which decreases under IR illumination similarly to peak 5 for Ce-doped crystals, but it is located at about 50 °C higher temperature.

### 3.3. Temperature Influence on IRSL

**Figure 13:**
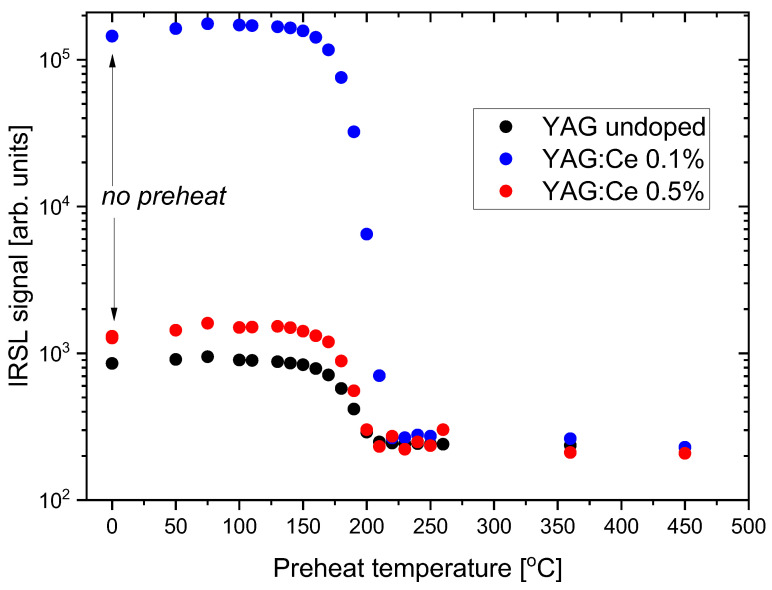
IRSL signal of YAG:Ce 0.1%, YAG:Ce 0.5 %, and undoped YAG crystals as a function of pre-heat temperature. The first data points represent no pre-heat. Dose 0.5 Gy.

Another way of obtaining some data about trapping sites responsible for the IRSL signal is to conduct a step-annealing experiment, i.e., to perform a series of IRSL measurements after pre-heating samples to increasing temperatures. The results of such measurements with the studied YAG crystals are presented in Figure 13. The pre-heat was carried out in the TL reader with heating to the set temperature at the rate of 5 °C/s.

It can be seen that the IRSL signal decreases in the temperature range 150−200 °C, i.e., at the temperatures corresponding with TL peaks P2 and P3, which agrees with the data from Figure 6, Figure 7, Figure 8, Figure 9, Figure 10, Figure 11 and Figure 12. There is also a small increase in IRSL intensity following pre-heat at low temperatures. The reason is probably a transfer of charge carriers from some shallow traps to traps responsible for IRSL. 

### 3.4. Emission Spectra

**Figure 14:**
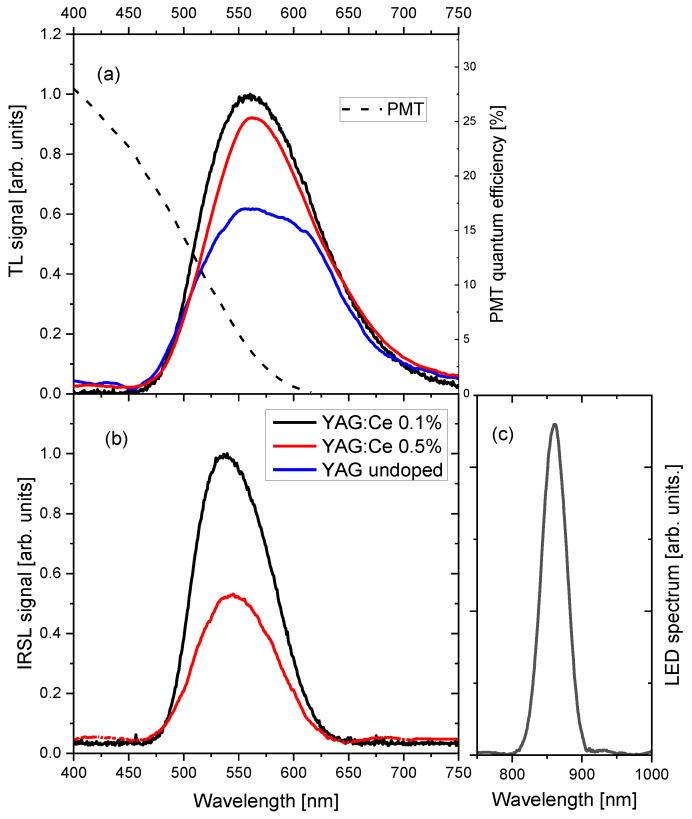
TL (**a**) and IRSL (**b**) emission spectra of the studied YAG crystals (dose 100 Gy). IRSL measurements performed with the application of the BG-39 filter. IRSL signal of the undoped YAG was too low to enable spectral measurements. The black dashed line in panel (**a**) illustrates the quantum efficiency of the photomultiplier of the DA-20 reader. Panel (**c**) presents the measured emission spectrum of LEDs used for the stimulation.

The TL and IRSL spectra are presented in Figure 14. The spectra, which peaked between 500 nm and 600 nm, have shapes typical for green-yellow emission of Ce^3+^ in garnet crystals, related to the 5d^1^→4f^2^ (^2^F_5/2,7/2_) transitions [20,26]. The fact that the spectrum of the undoped YAG crystal has also such a shape indicates that its luminescence is due to contamination of the nominally pure crystal with Ce^3+^ ions. The visible difference between TL and OSL spectra is caused by the application of the BG−39 filter, which was necessary to be used for IRSL measurement to suppress strong IR stimulation light. In the case of the undoped YAG, IRSL emission was too weak to be measured with the spectrometer. Comparing the measured spectra with the quantum efficiency of the photomultiplier of the DA-20 reader (Figure 14a), one can see that only a small part of the luminescence emission is measured using this reader. As most TL/OSL dosimetric materials emit in the UV/blue range, the readers are also optimized for this spectral range. If the Ce-doped garnets were to be used in dosimetric measurements, it would be therefore highly advantageous to apply measuring equipment with a better spectrally fitted light detection system.

### 3.5. Dosimetric Properties

This part of the investigation concerned practical aspects of exploiting IRSL of garnets for dosimetric measurements: stability of the signal over time and dose characteristics. In these measurements, we included for comparison purposes also the sample of GAGG:Ce crystal, which properties were described in the previous paper [9].

**Figure 15:**
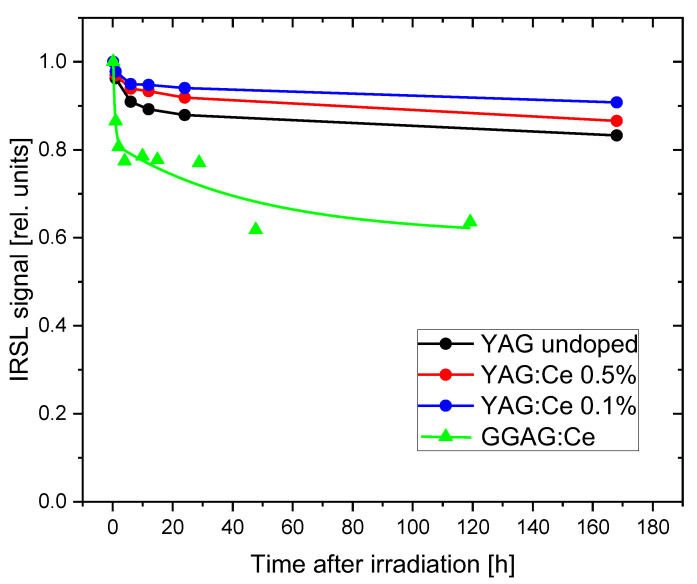
Fading of the IRSL signal during storage time after irradiation. The data are normalized to the first measurements, which were performed 10 min after the exposure. The lines are drawn only to guide the eye. Dose 1 Gy.

Figure 15 presents the data on the stability of the IRSL signal in the time following the irradiation. All YAG-based samples show a few percent decrease in the signal during the first hours after the exposure. For longer storage times the signal stabilizes. For the most sensitive crystal with 0.1% of Ce, we measured 91% of the initial signal after one week of storage, and the decay after the first 24 h was nearly negligible. The good stability exhibited by this crystal is quite remarkable and encouraging. There are several materials showing high OSL sensitivity, but is characterized by a high fading rate and are unsuitable for practical applications. This is probably the main reason that so far only two compounds—Al_2_O_3_:C and BeO, combining both properties: high sensitivity and low fading, found a successful way to implement in dosimetric systems.

The observed decrease of the signal during the first hours may be attributed to the decay of TL peaks located below and around 100 °C (peaks P0 and P1)—see Figure 6, Figure 7 and Figure 8, while the stable signal is related to peaks at higher temperatures. For GAGG:Ce, the fading of the IRSL signal is much stronger. The signal decreases to about 60%, but after 2 days it also seems to begin to stabilize.

**Figure 16:**
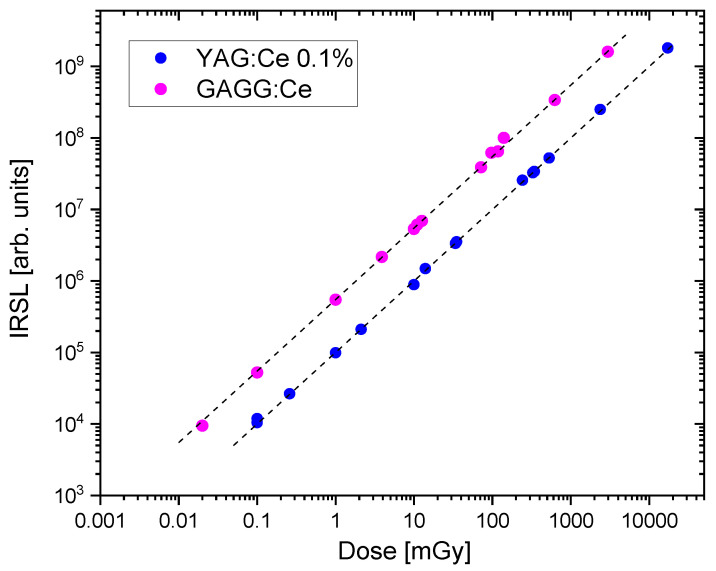
Dose-response for YAG:Ce 0.1% crystal, compared with that of GAGG:Ce crystal. The doses up to 10 mGy were delivered with ^137^Cs gamma-rays, while the higher doses with ^9^°Sr/^9^°Y beta-particles. Lines indicate linear trends.

**Figure 17:**
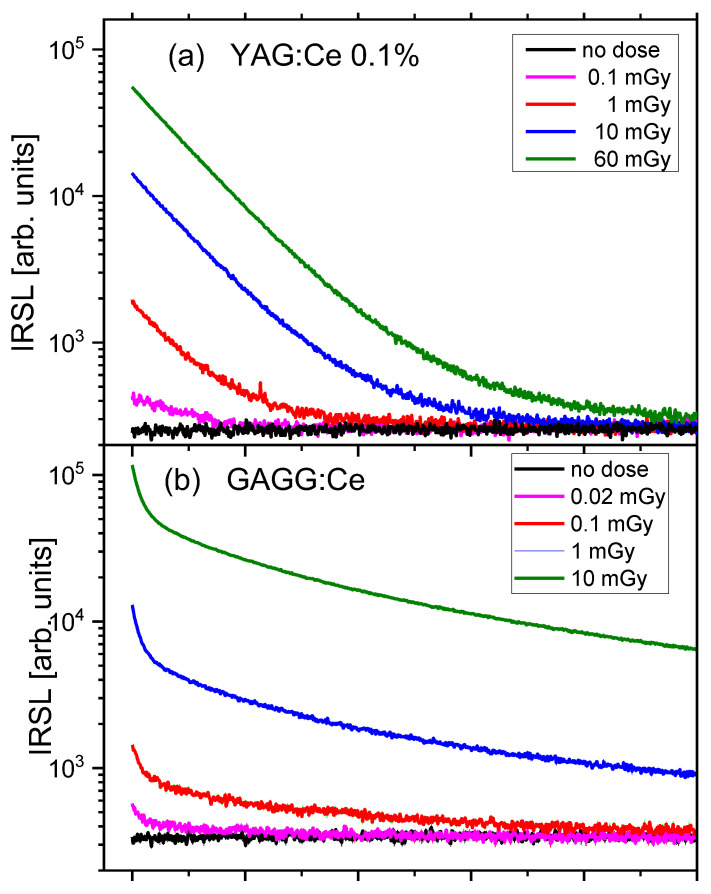
IRSL decay curves of (**a**) YAG:Ce 0.1% and (**b**) GAGG:Ce, corresponding to the lowest doses data points from Figure 16.

The dose response, i.e., the relationship between the IRSL signal and the delivered doses, was measured for the most sensitive YAG:Ce 0.1% crystal. The results, again in comparison with GAGG:Ce crystal, are presented in Figure 16. The data points follow a linear trend in the studied range of doses. It is remarkable that it was possible to measure even very low doses of 0.1 mGy for YAG:Ce crystal and 0.02 mGy for the more sensitive GAGG:Ce crystal. Figure 17 presents IRSL decay curves corresponding to the data from Figure 16, illustrating that the studied garnet crystals are indeed capable to produce meaningful IRSL signals, clearly exceeding the background level, even for these lowest doses. It should be emphasized, that these results were achieved by measuring only a part of the total emission, due to the mentioned limited spectral efficiency of the PMT at long wavelengths.

## 4. Concluding Remarks

In the present work, the infrared-stimulated luminescence of YAG:Ce crystals was investigated for the first time. All studied crystals exhibited bright IRSL emission, which was found to be related mainly to the TL peaks located between 150 °C and 200 °C. The intensity of IRSL highly depends on the content of Ce^3+^ dopant, as the sensitivity of the YAG crystal with 0.1% of Ce is nearly three orders of magnitude higher than that with 0.5%. 

These findings indicate the necessity of conducting more detailed investigations aimed at the optimization of the Ce concentration and this work is underway. It may be expected that such investigations will lead to a further increase in IRSL sensitivity of YAG:Ce crystal. Nevertheless, the sensitivity of the YAG:Ce 0.1% crystal is already fairly high, allowing measurements of the doses even at the level of 0.1 mGy. The high sensitivity combined with the relatively low fading creates good prospects for potential applications in dosimetric radiation measurements.

## Data Availability

Not applicable.
